# Implementation of a Physical Activity Program Protocol in Schoolchildren: Effects on the Endocrine Adipose Tissue and Cognitive Functions

**DOI:** 10.3389/fnut.2021.761213

**Published:** 2021-10-22

**Authors:** Javier Diaz-Castro, Jose Eulogio Garcia-Vega, Julio J. Ochoa, María Puche-Juarez, Juan M. Toledano, Jorge Moreno-Fernandez

**Affiliations:** ^1^Department of Physiology, Faculty of Pharmacy, Campus Universitario de Cartuja, University of Granada, Granada, Spain; ^2^Institute of Nutrition and Food Technology “José Mataix Verdú”, University of Granada, Granada, Spain; ^3^Instituto de Investigación Biosanitaria (IBS), Granada, Spain; ^4^Nutrition and Food Sciences Ph.D. Program, University of Granada, Granada, Spain

**Keywords:** children, physical activity, anthropometry, diet, adipose tissue, cognitive function

## Abstract

Practicing exercise is one of the best strategies to promote well-being and quality of life, however physical activity in schoolchildren and adolescents is developed in an unpredictable, intermittent way and in short periods. There are relatively few intervention studies investigating the role of physical activity in schoolchildren endocrine function of adipose tissue and cognitive function. One hundred and three boys, divided into two groups: control (*n* = 51, did not perform additional physical activity) and exercise (*n* = 52, performed vigorous physical activity after the regular school classes). The exercise group, developed a 6 months physical activity protocol delivered by the physical education teacher during the second semester of the academic course (6 months). Body composition measurements, adherence to the Mediterranean diet, nutritional intake, hematological and biochemical parameters, endocrine function of the adipose tissue and biomarkers of brain molecular function were assessed at enrolment and after 6 months of intervention. No statistically significant differences between both groups were found for age, height and bone mass. Weight and BMI was lower in the exercise group compared to the control group, increasing lean mass and reducing fat mass. 58.68% of children in the exercise group showed high adherence to the Mediterranean Diet compared to 46.32% of the control group. The exercise group was more concerned about their diet consumed more fiber, vitamin B1, B2, B6, B12, D, Niacin, Folic acid, Fe, Zn, Se and Cu. Triglycerides levels and HDL-cholesterol were higher in the exercise group at the end of the study. Leptin, MCP-1, lipocalin-2, adipsin and PAI-1 levels were lower in the exercise group at the end of the exercise protocol. In contrast, adiponectin and osteocrin markedly increased in the exercise group. Moreover, marked increases were recorded in healthy brain state biomarkers (NGF, BDNF, and irisin) in the exercise group, which could have a positive impact on academic performance. Taken together, all the findings reported are consistent with many benefits of the exercise protocol on adipose tissue and brain molecular function, demonstrating the usefulness of early interventions based on physical activity in children to reduce risk factors related to sedentary lifestyle.

## Introduction

Practicing exercise is one of the best strategies to promote well-being and quality of life (1). Specifically, systematic and regular physical activity contributes to maintaining and even improving different systems and functions, such as musculoskeletal, osteoarticular, cardiocirculatory, respiratory, endocrine-metabolic, immunological and psychoneurological functions (2). In addition, the improvement of some biomarkers, exercise not only positively affects physical health, but also mental health and quality of life (3, 4). Some studies have shown that patterns of habitual physical activity contribute to improving self-concept, self-esteem, depression and anxiety disorders (5, 6).

In general terms, physical activity in children is developed in an unpredictable, intermittent way and in short periods, which leads to affirm that its influence on health is underrated and even scarcely studied (7). Data from the European Youth Heart Study (8) suggested that the most active children (who dedicate at least 60 min/day of physical activity) have better cardiovascular health, regardless of their adiposity degree. This finding is noteworthy because there is a progressive increase in sedentary lifestyles related to the health problems of children, due to the misuse of free time, generally associated with the use of electronic devices (8–10).

Low levels of physical activity are related to overweight, increase of fat mass and obesity in schoolchildren and children (11) a period in which their practice tends to decrease (12). In addition, obesity has been linked to vascular endothelial dysfunction, which has been identified as a major risk factor for cognitive impairment (13). In this sense, some adipokines levels may be indicators of the effect that physical exercise could have on the endocrine function of adipose tissue. This is why their levels must be taken into account as they play a crucial role in the process. Thus, adiponectin which is an anti-inflammatory adipokine, regulates insulin homeostasis (14), leptin reduces food intake, increases energy expenditure and its levels are inversely correlated with adipose tissue (15), PAI-1 and MCP-1 regulate inflammatory signaling (16) and adipsin links the immune function and the adipose tissue endocrine function through the alternative pathway of complement activation (17), among others. Therefore, school age and adolescence, are crucial stages for the configuration of healthy lifestyle habits that will persist in later life stages (18). The development of unhealthy behavior patterns at these ages is a relatively generalized phenomenon and it is in them that adequate preventive measures have to be adopted that affect a better quality of life for the population (19).

In addition to promoting health and preventing disease, physical activity in schoolchildren is important for growth and development (20). Cognitive function, brain function, and learning outcomes have been reported to be linked to physical activity (21, 22). The association between physical activity, health and intelligence quotients has been shown in previous studies (23). This is why the assessment of some key parameters in cognitive function, such as NGF which enhances neurons differentiation and survival (24) and neurotransmitter biosynthesis, improving overall cognitive function (25), BDNF which has a key role on brain development, neuron proliferation and survival, and cognitive functions such as learning and memory (26, 27) and irisin which regulates the process of neuronal differentiation and maturation (28), is of high importance.

Moreover, there is a positive association between emotional quotient and physical activity (29). Higher emotional quotients have also been associated with longer exercise duration (30). Taken together, these studies suggest that increased physical activity is associated with higher intellectual and emotional cues during childhood. Despite the benefits described, several studies report that the practice of physical activity has suffered a progressive decline in young Spaniards, especially between 12 and 18 years of age (31), which is due by the increase of sedentary habits such as watching TV and playing video games (32). In this sense, there are relatively few intervention studies investigating the role of physical activity in schoolchildren on cognitive function. Furthermore, the results of these previous studies were mainly focused on body composition and psychological tests evaluating cognitive function, concentration, memory, and alertness without providing molecular mechanisms elucidating the role of physical activity in adipose tissue and brain functions. Hence, taking into account all these considerations, the purpose of this study was to determine the impact of a protocol of physical activity on children's endocrine function of adipose tissue and cognitive function biomarkers.

## Materials and Methods

### Subjects

A total of 122 students were asked to participate in the study. During the enrolment phase, 14 students refuse to participate, mainly because they were already performing sports extra-curricular activities several days per week after school hours, and one of them because he had a chronic disease (diabetes). Moreover, 5 students who agreed to participate in the study, finally left it because parents did not complete the informed consent form. The sample size selected for this study was 103 boys, divided into two groups using a simple randomization procedure (computerized random numbers) based on their initial weight to obtain statistically equal groups (ANOVA, *p* > 0.05). Control group, (*n* = 51, did not perform additional physical activity) and exercise group (*n* = 52, performed a more vigorous physical activity, according to the exercise protocol described below). The boys were studying during the second semester in a Center for Primary and Secondary Education in the Malaga region (Spain).

The mean age was 11.21 ± 0.17 years in the control group and 11.16 ± 0.18 years in the exercise group. The study was approved by the Ethics committee of Andalusia Biomedical Research Ethics Portal (ref. 29/01/2018/2/2018). Informed consent was obtained from all the parents with written consent to participate in this study. To avoid an important confounder in this type of trials we performed a 3 days diet questionnaire including 1 day of the weekend to assess the nutritional status of the participants. The information obtained in this survey was evaluated by nutritional software. A blood sample was obtained from each participant and they completed a medical and health history, physical activity questionnaire and anthropometric measurements.

### Calculation of Sample Size

According to earlier findings (33), a minimum sample size of 40 children per intervention is necessary to detect changes due to the physical activity across groups with a power of 80% and α = 0.05. As a result, a total of 80 subjects (40 per group) are required. A total of 103 schoolchildren were recruited to allow for a potential loss to follow-up of up to 25%.

### Physical Exercise Performance Protocol

**Figure 1:**
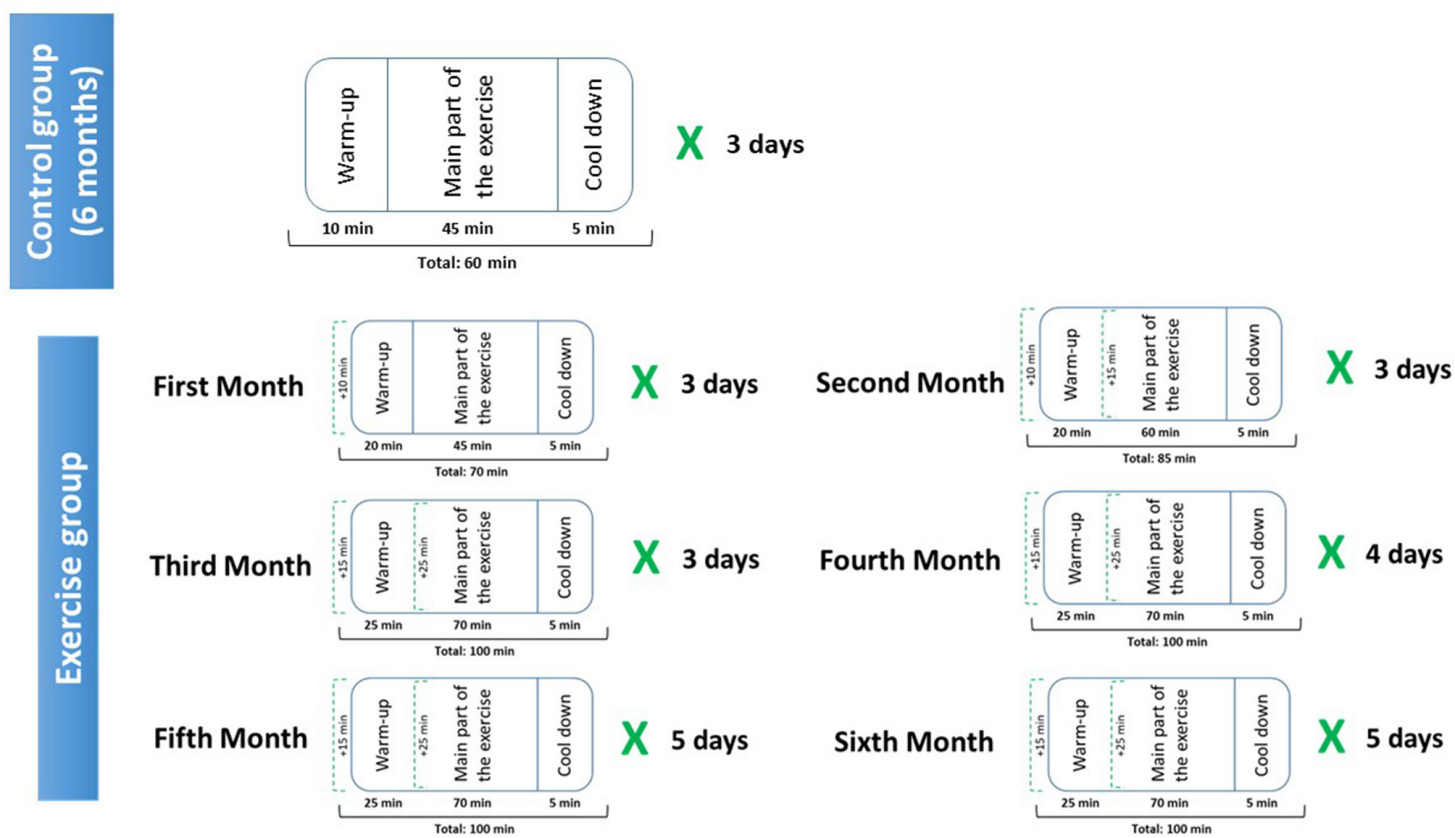
Physical exercise protocol.

The exercise protocol (Figure 1) was designed in accordance with a team of experts in sports and physical activity sciences. At the beginning of the study, all the participants (control and exercise groups), performed 3 days per week of training classes of 1 h that consists of three parts (A + B + C): (A) Warm-up (10 min): in which the boys begin with light-intensity movements (e.g., wrist rotations; leg swings). (B) Main part of the exercise (45 min): Technique exercises (15 min): passes, catches, drives, feints, dribbles, shots on goal, control exercises, skill circuits, tactic drills (15 min): rounds, defense drills, attack drills, counterattacks, set plays, superior attack, ball possession drills, pressures, field positions, lines, set pieces, real game situation “match” (15 min). (C) Cool down (5 min): stretching.

The intervention period was developed during the second semester of the academic course (from January to June). Control subjects continued with the routine mentioned above for the 6 months of the study, however, for the exercise group, the intervention consisted of a 6 months physical activity programme delivered by the physical education teacher with specific elements of additional vigorous physical activity, as follows:

- First month: 10 extra minutes of part A (warm up), of the protocol mentioned above are added, which means a total of 70 min of exercise protocol per day (3 days per week), which imp Total duration of the exercise protocol: 70 min. The teacher led an active 10-min warm-up, starting with light-intensity movements (e.g., wrist rotations; leg swings) and progressing through moderate-intensity activity (e.g., arm rotations; walking with a high knee-raise) to include two 4-min periods of vigorous physical activity (e.g., vigorous arm sprints; running on the spot). This warm-up evolves from mild to moderate-intensity activities and incorporates bursts of strong physical activity, which is designed to maximize the training benefit and to minimize the risk of injury. Five boys piloted the warm-up and infusions while wearing heart rate monitors on their chests, which demonstrated that heart rates were in the vigorous zone for the duration of all intervention elements. In total, we got an increase of 10 min of cardiovascular work per session (compared to previous sessions), which means 30 extra min per week, and about 120 min per month. The prescription was made based on the initial determination of the VO_2_ peak mL/kg/min and the clinical evaluation. VO_2_ peak was calculated with the 20 m shuttle run test using the protocol previously described by Léger et al. (34). The completed laps were noted when a child was unable to follow the pace in two consecutive intervals. For the following months, the volume was maintained with intensity variation (interval work, between 65% and 85% of the reserve heart rate) followed by resistance exercises with standard series.

- Second month: 25 min of aerobic work per session were added to the protocol developed in the first month, which means a total of 85 min of exercise protocol per day (3 days per week). 10 extra min to the part A (warm up) and 15 extra min to the part B (main part of the exercise).

-Third month: 15 min of aerobic work per session were added to the protocol developed in the second month, which means a total of 100 min of exercise protocol per day (3 days per week). 5 extra min to the part A (warm up) and 10 extra min to the part B (main part of the exercise).

- Fourth month: The duration and time of the exercise protocol (100 min per day), was followed as described on the third month, but one extra day per week was added to the exercise protocol (4 days per week) with the same characteristics mentioned in the third month.

- Fifth month and sixth month: The duration and time of the exercise protocol (100 min per day), was followed as described on the third and fourth months, but another one additional day per week was added to the exercise plan (5 days per week) during these 2 months, with the same characteristics mentioned in the third and fourth month.

### Blood Sampling

Blood samples were collected from the participants via venous catheter into heparinized tubes. Two blood samples were taken on each group: when enrolled in the study (T1, basal value), and after 6 months (T2). An aliquot od the blood was used for the measurement of hematological parameters and the rest of the blood was immediately centrifuged at 1,750 x g for 10 min at 4° C in a Beckman GS-6R refrigerated centrifuge (Beckman, Fullerton, CA, USA) to separate plasma from red blood cell pellets.

### Body Composition Measurements

The assessment of body composition was carried out by electrical bioimpedance (EIB), in the study a multifrequency TANITA MC-980MA equipment (Biológica Tecnología Médica S.L., Barcelona, Spain) with software Suite Biológica 7.1 (Version 368) was used. Following the guidelines supplied by the manufacturer, to carry out the measurement, the subjects were placed in an upright position on the platform and placing their bare feet on the four stainless steel electrodes in such a way that with the tip of the electrodes they made contact with the front electrodes and with the heel with the rear electrodes. The hands held the clamps with the electrodes, keeping the arms stretched along the trunk slightly apart from it. At no time during the test should there be contact with any metal part. An imperceptible multifrequency current between 5 and 1,000 kHz was introduced through the electrodes. The approximate measurement time was 30 s. Height was determined by the height contraction measurement method. The subjects were required to stand still with the feet together and the heels, buttocks, and upper back in contact with the scale. The parameters studied have been: weight, lean mass, fat mass, bone mass, and total body water. Body mass index (BMI) was calculated by dividing body weight in kilograms by height in meters squared (BMI = kg/m^2^) and height and weight were used to calculate age and gender-specific standardized child body mass index (BMI) (kg/m^2^) z-score.

### Physical Activity Measurement

The IPAQ-C questionnaire (35) designed to estimate physical activity in children aged 8–12 years was used, in which they asked about their physical activity during different times of the day, such as physical education class transport and free time to later find out the number of metabolic equivalents of task (MET) and determine the level of activity that a person has performed during the last 7 days. The children were classified into three categories (low, medium, high) according to the estimated energy expenditure for each activity: vigorous, 8 MET; moderate, 4 MET; and low, 3.3 MET. It consists of 10 questions that assess different aspects of the levels of physical activity performed by the child using the Likert scale to calculate the final score. MET-min per week, are obtained by multiplying energy expenditure for each activity by the minutes performed and by the days of the week you practice it.

### Adherence to the Mediterranean Diet

We assessed adherence to the Mediterranean dietary pattern by applying the KIDMED test of Adherence to the Mediterranean diet (MD) (36), used successfully in different populations (37), among them in Spanish children (36, 38). This instrument consists of 16 dichotomous questions that must be answered affirmatively/negatively (yes /no). The affirmative answers in the questions that represent a positive aspect in relation to the MD (12 questions) add one point, while the affirmative answers in the questions that represent a negative connotation with respect to the MD (four questions) subtract one point. Negative answers do not score. The total score obtained gives rise to the KIDMED index, which is classified into three categories:

a) From 0 to 3: Very low quality diet (low degree of adherence to MD).
b) From 4 to 7: Need to improve the eating pattern to adapt it to the MD (medium degree of adherence).
c) From 8 to 12: optimal MD (high degree of adherence).

### Nutritional Assessment

A 24-h recall questionnaire was carried out, it is a retrospective method to calculate the nutrient intake. The 24-h recall consists of asking the interviewed individual about the food consumed, both qualitatively and quantitatively, during a 24-h period. Three 24-h reminders were held including a holiday or a weekend day. The questionnaire was completed through a personal interview with a trained interviewer. To help the surveyed patient fill out this questionnaire and collect data as accurately as possible, a photographic manual was used that includes models of food sizes, prepared dishes, and homemade measurements (39). The duration of the survey was approximately 45 min.

### Software for Nutritional Assessment Processing

The data from the food consumption recall survey are processed through the Nutriber computer program (Nutriber, v1.1.1.5.r5, FUNIBER, Barcelona, Spain, 2005) (40), which allows us to know the amount of energy, macro and micronutrients consumed by the subjects and compare them with the recommended intakes for the Spanish population (41).

### Hematological Test

White blood cells (WBC), red blood cells (RBC), hemoglobin (Hb) concentration, mean corpuscular Hb (MCH), mean corpuscular Hb concentration (MCHC), haematocrit (HCT), platelets, lymphocytes, monocytes, neutrophils, eosinophils, basophils, mean corpuscular volume (MCV), mean platelet volume (MPV), red cell distribution width (RDW), plateletcrit (PCT) and platelets distribution width (PDW) of fresh blood samples were measured using an automated hematology analyzer Mythic 22CT (C2 Diagnostics, Grabels, France).

### Biochemical Parameters

Triglycerides, total cholesterol, HDL-cholesterol, LDL-cholesterol, uric acid, urea, albumin, creatine kinase-MB (CKMB), creatinine, aspartate transaminase (AST), alanine transaminase (ALT), alkaline phosphatase (AP), glucose, total bilirubin, total Protein and lactate dehydrogenase (LDH) were measured by standard colorimetric and enzymatic methods, using a BS-200 Chemistry Analyzer (Shenzhen Mindray Bio-Medical Electronics Co Ltd, Shenzhen, China). All of the samples were analyzed in duplicate, and the averages of the paired results were determined.

### Endocrine Function of the Adipose Tissue

Adiponectin, adipsin, lipocalin-2/NGAL, total plasminogen activator inhibitor-1 (PAI-1) and resisting were determined using the HADK1MAG-61K MILLIPLEX MAP Human Adipokine Magnetic Bead Panel 1 assay; leptin and monocyte chemoattractant protein-1 (MCP-1) levels were measured using the HADK2MAG-61K MILLIPLEX MAP Human Adipokine Magnetic Bead Panel 2 assay (Millipore Corporation, Missouri, USA); apelin, follistatin-like protein 1 (FSTL1), osteocrin and osteonectin were determined using the HMYOMAG-56K MILLIPLEX MAP Human Myokine Magnetic Bead Panel assay, based on immunoassays on the surface of fluorescent-coded beads (microspheres), following the specifications of the manufacturer (50 events per bead, 50 μl sample, gate settings: 8,000–15,000, time out 60 s). Plates were read on LABScan 100 analyzer (Luminex Corporation, Texas, USA) with xPONENT software for data acquisition. Average values for each set of duplicate samples or standards were within 15% of the mean. All the analytes in plasma samples were determined by comparing the mean of duplicate samples with the standard curve for each assay.

### Brain Molecular Function Parameters

Brain Derived Neurotrophic Factor (BDNF) and irisin were determined using the HMYOMAG-56K MILLIPLEX MAP assay (Millipore Corporation, Missouri, USA); Nerve growth factor (NGF) levels were measured using the HADK2MAG-61K MILLIPLEX MAP assay (Millipore Corporation, Missouri, USA). Plates were read as mentioned above.

### Statistical Analysis

All data are reported as mean values with their standard errors. All variables were tested to see if they followed the criteria of normality and homogeneity of variance using the Kolmogorov-Smirnoff's and Levene's tests, respectively. To compare general characteristics of the subjects in both experimental groups, unpaired Student's *t* test was used. Variance analysis by one-way ANOVA methods was used to compare the differences between periods. Following a significant F test (*P* < 0.05), individual means were tested by pair-wise comparison with Tukey's multiple comparison test, when main effects and interactions were significant. The level of significance was set at *P* < 0.05. Statistical analyses were performed using the SPSS computer program (version 26.0, 2021, SPSS Inc., Chicago, IL).

## Results

No statistically significant differences between both groups were found for age, height and bone mass. Weight, BMI and z-score were lower in the exercise group compared to the control group (*P* < 0.05) at the end of the intervention. Fat mass was drastically lower in the exercise group compared to the control group (*P* < 0.001) and the exercise group (*P* < 0.001) at the beginning of the study and the end of the exercise protocol. Lean mass and total water increased in the exercise group compared to the control group at the end of the study (*P* < 0.01) and also with the exercise group at the beginning of the study (*P* < 0.01). Finally, as expected, physical activity markedly increased in the exercise group compared to the control group (*P* < 0.001) after the exercise protocol and to the baseline with the exercise group (*P* < 0.001) (Table 1).

**Table 1:** Anthropometric characteristics and results of the International Physical Activity Questionary in the last 7 days from the control and exercise groups.

	**Control group**		**Exercise group**	
	**T1**	**T2**	**T1**	**T2**
Height (cm)	147.31 ± 1.76	148.01 ± 1.78	145.28 ± 2.09	146.35 ± 2.11
Weight (kg)	41.87 ± 1.88	42.65 ± 1.85^A^	41.95 ± 1.87	39.01 ± 1.86^B^^d^
Lean Mass (%)	45.65 ± 1.38	46.05 ± 1.61^A^	46.86 ± 1.45	53.37 ± 1.00^B^^d^
Fat Mass (%)	15.54 ± 1.56	16.25 ± 1.48^A^	15.21 ± 1.43	9.08 ± 0.87^B^^d^
Bone mass (%)	2.56 ± 0.11	2.59 ± 0.12	2.54 ± 0.12	2.51 ± 0.11
Total water (%)	58.66 ± 1.35	58.61 ± 1.42	58.73 ± 1.72	63.68 ± 1.88^d^
BMI (kg/m^2^)	20.66 ± 0.92	20.71 ± 0.91^A^	19.01 ± 0.87	18.18 ± 0.65^B^^d^
BMI (kg/m^2^) z-score	0.90 ± 0.01	0.94 ± 0.08^A^	0.93 ± 0.05	0.77 ± 0.03^B^^d^
Physical Activity (MET-min per week)	2,105.91 ± 132.05	1,980.32 ± 127.31	2,117.23 ± 115.59	3,387.85 ± 129.33^B^^d^

*Mean values among groups with different letters differ (P < 0.05)*.

A,B *for T2 by Student's t test between different groups;*

d *for T1 vs. T2 in the same group by Tukey's test*.

Table 2 shows that 58.68% of children in the exercise group showed high adherence to the Mediterranean diet compared to 46.32% of the control group at the end of the intervention (*P* < 0.05).

**Table 2:** Results of the KIDMED test evaluating the adherence to the Mediterranean diet from the control and exercise groups.

	**Control group**		**Exercise group**	
	**T1**	**T2**	**T1**	**T2**
Low (≤3, %)	0	0	0	0
Medium (4–7, %)	54.73 ± 0.30	53.68 ± 0.33^A^	53.25 ± 0.29	41.32 ± 0.39^B^^d^
High (≥8, %)	45.27 ± 0.27	46.32 ± 0.32^A^	46.75 ± 0.28	58.68 ± 0.34^B^^d^
Mean Value	6.82 ± 0.31	6.25 ± 0.27^A^	6.85 ± 0.31	7.82 ± 0.37^B^^d^

*Mean values among groups with different letters differ (P < 0.05)*.

A,B *for T2 by Student's t test between different groups;*

d *for T1 vs. T2 in the same group by Tukey's test*.

Regarding energy intake and most of the macronutrients, no statistically significant differences between both groups were found, however the exercise group consumed more fiber (*P* < 0.05), vitamin B1 (*P* < 0.01), B2 (*P* < 0.01), B6 (*P* < 0.01), B12 (*P* < 0.05), D (*P* < 0.01), Niacin (*P* < 0.001) and Folic acid (*P* < 0.01) compared to the control group and also to the same group at enrolment. With regards to minerals, the exercise group consumed more Fe (*P* < 0.001), Zn (*P* < 0.05), Se (*P* < 0.01) and Cu (*P* < 0.01) compared to the control group and also to the same group at enrolment (Table 3).

**Table 3:** Diet composition and nutritional intake analyses from the control and exercise groups.

	**Control group**		**Exercise group**	
	**T1**	**T2**	**T1**	**T2**
Energy (Kcal)	1,864.07 ± 50.76	1,935.12 ± 60.15	1,889.45 ± 63.28	1,945.52 ± 63.38
Carbohydrates (%)	45.74 ± 1.25	46.21 ± 1.87	45.39 ± 1.76	45.43 ± 1.29
Protein (%)	17.29 ± 0.53	17.75 ± 0.59	17.11 ± 0.49	17.08 ± 0.55
Lipids (%)	36.97 ± 1.01	37.43 ± 1.09	36.88 ± 1.07	37.42 ± 1.06
Fibre (g)	12.21 ± 0.70	11.97 ± 0.73^A^	12.02 ± 0.68	13.93 ± 0.51^B^^d^
Vitamin A (μg)	1,114.32 ± 97.60	1,075.27 ± 101.07^A^	1,121.32 ± 96.51	1,167.78 ± 118.31
Vitamin B_1_ (mg)	1.42 ± 0.11	1.39 ± 0.21^A^	1.47 ± 0.23	1.90 ± 0.19^B^^d^
Vitamin B_2_ (mg)	1.25 ± 0.10	1.33 ± 0.14^A^	1.29 ± 0.12	1.62 ± 0.23^B^^d^
Vitamin B_6_ (mg)	1.42 ± 0.12	1.49 ± 0.13^A^	1.48 ± 0.14	1.89 ± 0.35^B^^d^
Vitamin B_12_ (μg)	5.26 ± 0.98	4.97 ± 0.87^A^	5.01 ± 0.94	5.91 ± 0.36^B^^d^
Vitamin E (μg)	6.74 ± 0.41	6.32 ± 0.39	6.41 ± 0.49	6.62 ± 0.53
Vitamin C (mg)	110.58 ± 12.96	115.27 ± 11.87	108.32 ± 10.35	107.25 ± 10.11
Vitamin D (μg)	5.92 ± 0.71	5.76 ± 0.65^A^	5.87 ± 0.39	6.91 ± 0.35^B^^d^
Niacin (mg)	16.74 ± 1.21	17.43 ± 1.03^A^	16.65 ± 1.23	20.21 ± 1.89^B^^d^
Folic Acid (μg)	157.34 ± 17.86	153.45 ± 16.45	158.45 ± 18.31	178.76 ± 16.45^B^^d^
P (mg)	993.77 ± 39.23	1,001.27 ± 41.40	998.98 ± 39.89	1,006.36 ± 49.22
Mg (mg)	160.58 ± 7.97	163.67 ± 6.69	164.39 ± 7.01	165.31 ± 8.05
Ca (mg)	757.34 ± 37.24	755.11 ± 33.23	760.21 ± 31.54	751.98 ± 35.28
Fe (mg)	10.11 ± 0.68	10.15 ± 0.73^A^	10.98 ± 0.81	12.52 ± 0.71^B^^d^
Na (mg)	1,496.65 ± 119.21	1,503.98 ± 105.22	1,505.42 ± 99.87	1,591.06 ± 101.89
K (mg)	1,582.65 ± 63.97	1,598.43 ± 77.13	1,601.11 ± 65.34	1,688.65 ± 116.25
Zn (mg)	6.37 ± 0.31	6.22 ± 0.41^A^	6.12 ± 0.29	7.68 ± 0.32^B^^d^
Se (μg)	57.21 ± 6.11	59.22 ± 5.98^A^	62.25 ± 4.43	75.25 ± 8.39^B^^d^
F (μg)	284.33 ± 21.12	297.22 ± 22.75	289.45 ± 22.16	301.17 ± 34.25
I (μg)	48.38 ± 4.45	47.45 ± 4.12	46.98 ± 4.01	47.66 ± 6.56
Cu (μg)	672.21 ± 71.30	681.32 ± 68.85^A^	701.32 ± 70.23	944.35 ± 88.32^B^^d^

*Mean values among groups with different letters differ (P < 0.05)*.

A,B *for T2 by Student's t test between different groups;*

d *for T1 vs. T2 in the same group by Tukey's test*.

With regards to most of the hematological parameters studied, no statistically significant differences between both groups were found except for the increased in WBC recorded for the experimental group compared to the control group at the end of the study (*P* < 0.05) and RDW which was higher in both groups at the end of the study (*P* < 0.01) (Table 4).

**Table 4:** Hematological parameters from the control and exercise groups.

	**Control group**		**Exercise group**	
	**T1**	**T2**	**T1**	**T2**
WBC (10^3^/μL)	5.91 ± 0.28	6.05 ± 0.23^A^	6.01 ± 0.31	6.73 ± 0.32^B^^d^
RBC (10^6^/μL)	4.59 ± 0.03	4.65 ± 0.04	4.75 ± 0.07	4.84 ± 0.08
Hb concentration (g/dL)	13.73 ± 0.19	13.95 ± 0.13	14.06 ± 0.07	14.18 ± 0.22
MCH (g/dL)	29.81 ± 0.27	29.99 ± 0.32	29.83 ± 0.29	29.58 ± 0.28
MCHC (g/dL)	36.38 ± 0.35	37.83 ± 0.24	36.23 ± 0.34	37.72 ± 0.22
HCT (%)	36.83 ± 0.64	36.98 ± 0.39	37.11 ± 0.53	37.81 ± 0.65
Platelets (10^3^/μL)	305.12 ± 10.55	309.12 ± 13.54	289.22 ± 14.21	308.11 ± 13.55
Lymphocytes (10^3^/μL)	2.33 ± 0.10	2.35 ± 0.10	2.45 ± 0.12	2.44 ± 0.13
Monocytes (10^3^/μL)	0.36 ± 0.02	0.32 ± 0.02	0.37 ± 0.03	0.33 ± 0.02
Neutrophils (10^3^/μL)	2.87 ± 0.26	2.91 ± 0.22	2.83 ± 0.18	2.85 ± 0.12
Eosinophils (10^3^/μL)	0.33 ± 0.06	0.35 ± 0.09	0.32 ± 0.02	0.31 ± 0.02
Basophils (10^3^/μL)	0.03 ± 0.01	0.02 ± 0.01	0.02 ± 0.01	0.02 ± 0.01
MCV (fL)	80.40 ± 1.25	79.29 ± 0.88	80.28 ± 0.89	79.41 ± 0.88
MPV (fL)	8.69 ± 0.14	9.23 ± 0.16	8.21 ± 0.17	8.96 ± 0.19
RDW (%)	14.26 ± 0.28^a^	16.11 ± 0.25^d^	13.32 ± 0.28^b^	16.26 ± 0.16^d^
PCT (%)	0.26 ± 0.01	0.28 ± 0.01	0.24 ± 0.01	0.25 ± 0.01
PDW (%)	13.46 ± 0.26	14.01 ± 0.30	13.67 ± 0.22	14.11 ± 0.33

*Mean values among groups with different letters differ (P < 0.05)*.

a,b *for T1 and*

A,B *for T2 by Student's t test between different groups;*

d *for T1 vs. T2 in the same group by Tukey's test*.

Biochemical parameters are shown in Table 5. Triglycerides levels were lower in the exercise group compared to the control group at the end of the study (*P* < 0.01) and, in contrast, they increased in both groups after the 6 months period (*P* < 0.001 for the control group and *P* < 0.01 for the exercise group). In the control group HDL-cholesterol decreased after the 6 months period (*P* < 0.001) and, in contrast, in the exercise group increased after the exercise intervention (*P* < 0.01). AP increased in the exercise group compared to the control group after the physical activity intervention (*P* < 0.001). AP also increased in both groups at the end of the 6 months intervention, compared to the beginning of the study (*P* < 0.001).

**Table 5:** Biochemical parameters from the control and exercise groups.

	**Control group**		**Exercise group**	
	**T1**	**T2**	**T1**	**T2**
Triglycerides (mg/dL)	71.98 ± 10.29	139.36 ± 13.29^A^^d^	69.84 ± 8.71	98.55 ± 11.84^B^^d^
Total cholesterol (mg/dL)	153.01 ± 7.25	151.55 ± 7.57	156.06 ± 8.16	153.87 ± 5.79
HDL-cholesterol (mg/dL)	71.45 ± 5.61	68.43 ± 3.81^A^^d^	73.29 ± 3.32	83.27 ± 4.01^B^^d^
LDL-cholesterol (mg/dL)	32.41 ± 2.22	33.54 ± 3.29	32.54 ± 2.24	31.42 ± 1.92
Uric Acid (mg/dL)	5.37 ± 0.55	5.11 ± 0.40	4.12 ± 0.45	4.19 ± 0.28
Urea (mg/dL)	27.20 ± 2.47	28.00 ± 1.39	28.11 ± 1.93	27.05 ± 3.22
Albumin (g/dL)	4.27 ± 0.07	4.12 ± 0.06	4.25 ± 0.04	4.07 ± 0.02
CK-MB (U/L)	52.64 ± 2.68	37.01 ± 1.99	52.13 ± 2.13	37.24 ± 1.29
Creatinine (mg/dL)	0.62 ± 0.03	0.79 ± 0.03	0.68 ± 0.05	0.77 ± 0.03
AST (U/L)	27.96 ± 2.27	27.13 ± 1.26	27.56 ± 2.01	27.52 ± 1.58
ALT (U/L)	21.57 ± 2.36	24.79 ± 2.36	22.27 ± 2.09	22.91 ± 2.35
AP (U/L)	143.29 ± 25.21	232.98 ± 26.41^A^^d^	144.39 ± 27.20	311.61 ± 29.24^B^^d^
Glucose (mg/dL)	95.03 ± 3.61	95.33 ± 2.88	96.95 ± 3.25	95.32 ± 3.30
Total bilirrubin (mg/dL)	2.05 ± 0.14	1.93 ± 0.09	2.08 ± 0.24	2.11 ± 0.05
Total Protein (g/dL)	8.02 ± 0.16	7.98 ± 00.6	7.83 ± 0.15	8.07 ± 0.23
LDH (U/L)	459.13 ± 27.53	445.11 ± 15.91	446.43 ± 21.33	438.87 ± 22.23

*Mean values among groups with different letters differ (P < 0.05)*.

A,B *for T2 by Student's t test between different groups;*

d *for T1 vs. T2 in the same group by Tukey's test*.

**Figure 2:**
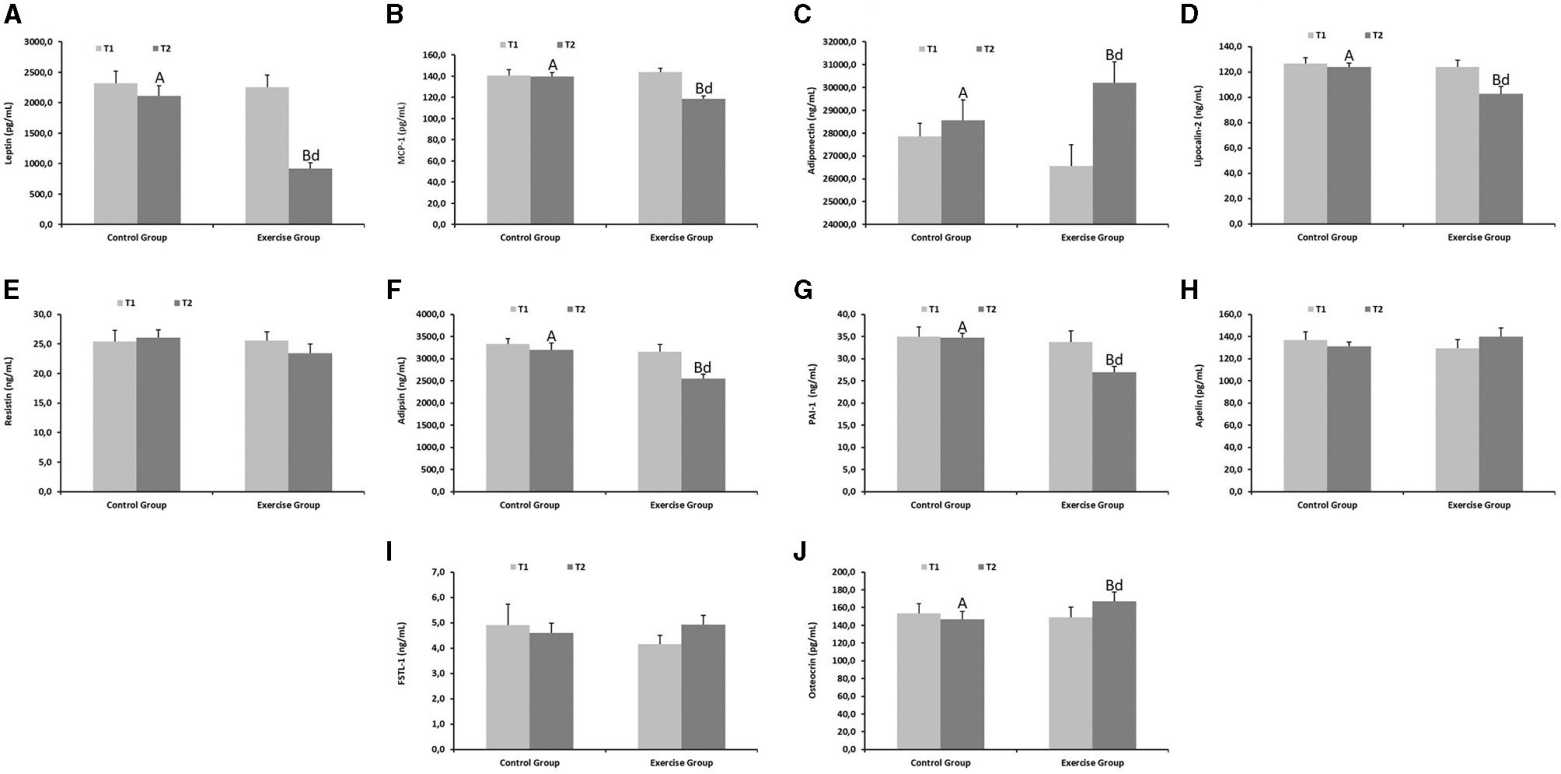
Endocrine function parameters of the adipose tissue from the control and exercise groups. Mean values among groups with different letters differ (*P* < 0.05) (^a,b^for T1 and ^A,B^for T2 by Student's *t* test between different groups; ^d^for T1 *vs*. T2 in the same group by Tukey's test). **(A)** Leptin, **(B)** MCP-1, **(C)** Adiponectin, **(D)** Lipocalin-2, **(E)** Resistin, **(F)** Adipsin, **(G)** PAI-1, **(H)** Apelin, **(I)** FSTL-1, **(J)** Osteocrin. MCP-1, Monocyte Chemoattractant Protein-1; PAI-1, Plasminogen activator inhibitor-1; FSLT-1, Follistatin-related protein 1.

Endocrine function parameters of the adipose tissue are shown in Figure 2. Leptin (Figure 2A), MCP-1 (Figure 2B), lipocalin-2 (Figure 2D), adipsin (Figure 2F) and PAI-1 (Figure 2G) levels were lower in the exercise group compared to the control group at the end of the exercise protocol (*P* < 0.001 for leptin, *P* < 0.01 for MCP-1; *P* < 0.01 for lipocalin-2; *P* < 0.001 for adipsin; *P* < 0.001 for PAI-1). In contrast, adiponectin (Figure 2C) and osteocrin (Figure 2J) markedly increased in the exercise group at the end of the study (*P* < 0.01).

**Figure 3:**
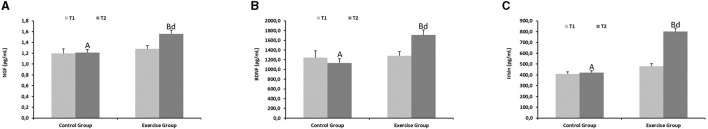
Cognitive function parameters from the control and exercise groups. Mean values among groups with different letters differ (*P* < 0.05) (^a,b^for T1 and ^A,B^for T2 by Student's *t* test between different groups; ^d^for T1 *vs*. T2 in the same group by Tukey's test). NGF, Nerve growth factor; BDNF, Brain-derived neurotrophic factor. **(A)** NGF, **(B)** BDNF, **(C)** Irisin.

Brain cognitive function biomarkers showed marked increases in the exercise group compared to the controls at the end of the physical activity protocol (Figure 3) (*P* < 0.01 for NGF, Figure 3A; *P* < 0.001 for BDNF, Figure 3B and *P* < 0.001 for irisin, Figure 3C).

Finally, no statistically significant differences between both groups were found for Natural Sciences, Arts, Social Sciences and Language qualifications, meanwhile Maths and Physical education qualifications were higher in the exercise group compared to the controls at the end of the semester (*P* < 0.05 for Maths; *P* < 0.01 for physical education) (Table 6).

**Table 6:** Academic qualifications from the control and exercise groups at the end of the semester.

	**Control group**	**Exercise group**
Maths	6.76 ± 0.42^a^	7.85 ± 0.55^b^
Arts	7.57 ± 0.45	7.26 ± 0.47
Language	7.38 ± 0.41	7.58 ± 0.49
Natural sciences	7.53 ± 0.51	7.66 ± 0.52
Social sciences	7.00 ± 0.65	7.08 ± 0.66
Physical education	7.61 ± 0.36^a^	8.58 ± 0.28^b^

*Mean value ± SEM for ten rats per group*.

a,b *Mean values among groups with different letters differ (P < 0.05) (by Student's t test)*.

## Discussion

Previously, several interventions have been carried out to promote physical activity in schools (42–46), however no previous study has examined the influence of exercise on the endocrine adipose tissue and cognitive function biomarkers in young schoolchildren/adolescents as the current study does. In this sense, the current research has several advantages. The physical activity protocol was designed to maximize the benefits of training and minimize the risk of injury, being low cost and really easy to implement with scarce equipment and facilities. By involving teachers in the design and implementation of the protocol, enables them to acquire the knowledge and skills to design and implement protocols for other students and groups, facilitating additional activities development which will contribute to promote physical activity at school.

Overall, the findings indicate that the although children were already active before the exercise protocol, it is encouraging that a marked significant increase in physical activity was observed in the intervention group from baseline to 6 months follow-up, particularly as this is an age when physical activity participation tend to decrease dramatically (47). The findings of the current study are in agreement with a previous report indicating that exercise improves BMI, increasing lean mass and reducing fat mass in children and adolescents (48).

In the current study, 58.68% of children in the exercise group showed high adherence to the Mediterranean Diet compared to 46.32% of the control group at the end of the physical activity intervention, reflecting those children who practice more physical activity are also more concerned about their diet, which is in agreement with previous results from other studies (37, 38). In addition, Kontogianni et al. (49) also reported that the time dedicated to sedentary activities was lower as the degree of adherence to Mediterranean Diet increased in children and adolescents (3–18 years). Most of the published studies that include Spanish children show a percentage of subjects with adherence to Mediterranean Diet similar to the control group, thus Serra-Majem et al. (50), reported that 48.5% of individuals aged 2–14 years and 44.6% of those aged 15–24 years followed an optimal Mediterranean Diet. Mariscal-Arcas et al. (51), found high adherence to Mediterranean Diet in 46.9% of the subjects aged 8–9 years and in 48.9% of children aged 10–16 years. Ayechu and Durá (2009), found an optimal Mediterranean Diet in 42.9% of 13–16 years old. Rodríguez et al. (52), found a high adherence to the Mediterranean dietary pattern in 42.8% of the children included in the study.

Although total dietary intake patterns may not promote cognitive function during aging (for example, the Western Diet is associated with accelerated aging of the brain), however diet may be beneficial (in this sense, Mediterranean Diet is associated with delayed brain aging and improved cognitive ability) (53). In the current study, the exercise group consumed more fiber which is typically found in many foods of the Mediterranean diet, influencing the relationship between diet and learning through the establishment and maintenance of the gut microbiome by establishing beneficial bacteria in the colon of the human digestive system. The potential role of the “gut-brain axis” in the human body is believed to be fundamentally important. But so far, only animal studies have revealed the possible directions and extent of this relationship (54).

Due to the higher adherence to the Mediterranean Diet, the exercise group consumed more vitamin B complex, vitamin D, folate, Cu, Zn, Se, and Fe. Schoolchildren nutritional status, especially the adequacy of nutrients such as Fe, is positively correlated with various measures of cognitive ability (55). Supplementing children's nutrition with Fe offers many cognitive benefits, including improved working memory, school attendance and learning experience (56). Another micronutrient important for brain development and function in school-aged children is folic acid (57), which is positively associated with academic performance. There is also evidence that vitamin B complex influences memory function and cognitive outcomes, because they are involved in the formation of synapses, the growth of axons and myelin genesis. In this sense, vitamin B12 is important for cognitive development (58). An association between vitamin B12 status and cognitive function has also been documented from childhood to adolescence. Children fed on a macrobiotic diet low in vitamin B12 featured delayed overall motor, speech, and language development compared with children on an omnivorous diet (59). In adolescence, these children who were fed a low vitamin B12 diet for the first 6 years had lower cognitive test scores than children who were fed an omnivorous diet (60). Vitamin D deficiency is well known to be associated with cognitive impairment because it is involved in synaptic plasticity, which regulates brain plasticity by interacting with the synaptic network aggregates of the extracellular matrix (61). Zn is necessary for normal brain development and plays an essential role in neuronal migration, neurite formation and synapse formation (62). Although limited, there is some evidence that Zn integration provides better attention and abstract reasoning (56, 63). Zn deficiency is associated with increased rates of neurodevelopmental disorders and attention deficit hyperactivity disorder (54). Dietary zinc, copper, and selenium intake is inversely associated with low cognitive performance in an L-shaped dose–response relationship (64), therefore the higher intake of these minerals in the exercise group might help to the better cognitive performance of the children.

With regard to the hematological parameters, the most outstanding result recorded in the current study was a slight leukocytosis in the exercise group after the physical activity intervention, which can be explained because of the catecholamine-induced demarginating of white blood cells and increased release from the bone marrow storage reserve during exercise (65). AP increased in both groups after the 6 months follow-up, reflecting the rapid growing peak of the children and increased bone metabolism (66). In addition, AP also increased in the exercise group compared to the control group, because AP is involved in ATP metabolism during exercise, by the participation in amino acid catabolism and the fat hydrolysis trough cell membranes (67).

Previous research has established cross-sectional links between adipose tissue and cognition in preadolescent children, although many of these have been conducted in obese or overweight subjects (68–72), without elucidating the role of the endocrine function of the adipose tissue and the molecular mechanisms involved in brain functions. Our study extends this knowledge by investigating the role of adipokines and the relationship with some molecular function biomarkers in normal weight schoolchildren to establish a relationship between changes in adiposity, physical activity and cognitive function after a physical activity program.

Adiponectin is a potent anti-inflammatory adipokine which also regulates insulin homeostasis, reducing the risk of many chronic diseases, including atherosclerosis, hypertension, nonalcoholic fatty liver, metabolic syndrome, cardiovascular disease, thrombosis, and asthma (14). Leptin reduces food intake, increases energy expenditure and its levels are inversely correlated with adipose tissue (15). In the present study, we have found a reduction in leptin and an increase in adiponectin after the exercise protocol, which is consistent with previous studies of physical activity in children (73). The study conducted by Many et al. (74) reported a decrease in leptin values after physical exercise, although 55% of the subjects continued to have high values when compared with the control subjects, which is explained because in this study they included obese and overweight children. In our study, no weight reduction was found, but a marked decrease in fat mass, which may explain the reduction in leptin and the increase in adiponectin compared to the control group at the end of the exercise protocol, reducing the risk of inflammation and insulin resistance (73, 75, 76). In the current study, the group that performed physical activity featured an improvement in the lipid profile, being the decrease in triglycerides and the increase in HDL-cholesterol statistically significant, which is in agreement with a previous study carried out in children performing aerobic exercise for 8 months (77).

Accumulating evidence supports the belief that exercise can reduce chronic inflammatory response, thus preventing the development of chronic diseases (78). Therefore, exercise may protect nervous system via its anti-inflammatory effects, which is one of the main reasons for the improvement of cognitive function (79). Adipose tissue endocrine function changed profoundly in the exercise group. In our study a lower level of MCP-1 was found in the exercise group, which may explain, at least in part, the reason for their improvement in brain molecular functions. PAI-1regulates several processes, including cell death, senescence, inflammation and it has been identified as key biomarker of several pathological conditions (16). Control of PAI-1 levels and several studies indicate that PAI-1 can be controlled with exercise (80–82), as we have recorded in the current study. Lipocalin-2 is as a pleiotropic molecule involved in a variety of physiological and pathological processes, such as metabolic homeostasis, apoptosis, infection, immune response, or inflammation (83). As previously reported (84), the reduction in lipocalin-2 due to the exercise is linked with other phenomena, like reduction of inflammatory markers, as we also recorded in the current study in the exercise group. Adipsin is an adipokine which links the immune function and the adipose tissue endocrine function, through the alternative pathway of complement activation (17). Our results are in agreement with previous results, indicating that exercise decreases adipsin levels (85). Osteocrin is a molecule released when performing physical activity which improves neuronal function, bone development, and physical endurance (86), prevents inflammatory signaling, cardiac rupture, and heart failure (87). In our study, osteocrin was also increased in the exercise group revealing the intensity of the exercise protocol and additional benefits of the physical activity in the children.

With regard to the brain molecular functions studied, NGF is highly expressed in developing hippocampus and cerebral cortex (88), enhances cholinergic neurons differentiation and survival (24), acetylcholine biosynthesis as well as activity and expression of cholinergic markers, improving overall cognitive function (25). As previously reported, exercise increases NGF production (89), results in agreement with those obtained in the current study. One of the most outstanding results is the increase in the BDNF levels in the exercise group. BDNF is a neurotrophin with a key role on brain development, neuron proliferation and survival, and for cognitive functions such as learning and memory (26, 27). During exercise, skeletal muscle increases expression of hippocampal BDNF (28). In particular, muscle contraction cleaves type III domain-containing protein 5, a protein secreted from the sarcolemma into the bloodstream as irisin. Then, irisin stimulates BDNF expression in the hippocampus and regulates the process of neuronal differentiation and maturation (28). Increased levels of BDNF in the exercise group is an essential mechanism explaining the exercise-induced brain plasticity and cognitive functions enhancement (90–93), revealing a better healthy brain state and a good predisposition for an improvement in cognitive function, which may be influenced by multiple factors, so that it should be confirmed in further studies with longer duration and sample size, as well as together with other biomarkers of cognitive function and some test that allow to better assesses the improvement in the cognitive development.

Our study has also shown differences in the academic performance of children, assessed as part of the academic regular evaluations (papers, exams and assessments performed by the respective teachers of each subject) during the academic course in the school, that could be due to physical activity. These results are in agreement with those obtained previously, in which increased lean mass, decreased fat mass and the amount of weekly physical activity are related to greater cognitive competence in both children and adolescents, especially in Maths (94–98). In this sense, practicing daily physical exercise of moderate intensity with a duration of between 10 and 50 min (98), would be enough to increase academic performance and cognitive performance (94, 99). Taking into account the positive effects of NGF, BDNF, and irisin on the brain and their functional implications, it is really interesting to promote exercise protocols for maximizing circulating levels of these neurotrophins during the school age, which would have a positive impact on academic performance. Taken together, all the findings reported in the current study are consistent with many benefits of the exercise protocol on adipose tissue and brain molecular function.

This study features some strengths and limitations that should be taken into account. Regarding to the strengths, this study proposes the development of a new physical activity protocol. The physical activity protocol was designed to maximize the benefits of training and minimize the risk of injury, being low cost and really easy to implement with scarce equipment and facilities. By involving teachers in the design and implementation of the protocol, enables them to acquire the knowledge and skills to implement this protocol for other students and groups, facilitating additional activities development which will contribute to promote physical activity at school. Moreover, to our knowledge, this is the first time, that the relationship between adipose tissue endocrine function and cognitive function in physically active school children has been assessed. Finally, this exercise protocol could help to improve the perception of the importance of a healthy lifestyle at a stage of life in which habits are created that last over time. Under the heading of limitations, the following should be noted; the sample size could be larger as well as the duration of the study should be prolonged to a full academic year and not just one semester, in order to observe adipose tissue endocrine and cognitive functions changes in the long term. In addition, the study could have included another sub-population made up of girls to see the influence of physical activity on this group, which is more reluctant to do it, and more cognitive parameters and some test that assesses performance and IQ before and after the physical exercise protocol, should have been measured.

## Conclusions

Overall, the findings indicate a marked increase in physical activity in the intervention group, which is particularly interesting as this is an age when physical activity participation tend to decrease dramatically. At the end of the physical activity intervention, the children were also more concerned about their diet, and due to the higher adherence to the Mediterranean Diet, the exercise group consumed more vitamin B complex, vitamin D, folate, Cu, Zn, Se, and Fe which might help to improve cognitive performance of the children. In our study, no weight reduction was found, but improved BMI and z-score, increasing lean mass and reducing fat mass, which may explain the reduction in leptin and the increase in adiponectin compared to the control group at the end of the exercise protocol, reducing the risk of inflammation and insulin resistance, together with an improvement in the lipid profile. The exercise protocol also reduced inflammatory response, which could also contribute to the improvement of the healthy brain state. Finally, exercise increased NGF, BDNF, and irisin, and taking into account their roles on the brain and their functional implications, it is really interesting to promote exercise protocols for maximizing circulating levels of these neurotrophins during the school age, which would have a positive impact on academic performance. Taken together, all the findings reported in the current study are consistent with many benefits of the exercise protocol on adipose tissue and brain molecular function, demonstrating the usefulness of early interventions based on physical activity in children to reduce risk factors related to sedentary lifestyle.

## Data Availability Statement

The raw data supporting the conclusions of this article will be made available by the authors, without undue reservation.

## Ethics Statement

The studies involving human participants were reviewed and approved by Ethics Committee of Andalusia Biomedical Research Ethics Portal (ref. 29/01/2018/2/2018). Written informed consent to participate in this study was provided by the participants' legal guardian/next of kin.

## Author Contributions

JD-C, JM-F, and JG-V performed the experiments, wrote, and prepared the original draft. JO analyzed and summarized data. MP-J and JT contributed to the data acquisition and critically reviewed the manuscript. JD-C and JM-F supervised project administration, provided resources, funding, and reviewed the manuscript. All authors contributed to the article and approved the submitted version.

## Funding

JM-F was supported by a Postdoctoral Contract (Perfeccionamiento de Doctores) from the University of Granada.

## Conflict of Interest

The authors declare that the research was conducted in the absence of any commercial or financial relationships that could be construed as a potential conflict of interest.

## Publisher's Note

All claims expressed in this article are solely those of the authors and do not necessarily represent those of their affiliated organizations, or those of the publisher, the editors and the reviewers. Any product that may be evaluated in this article, or claim that may be made by its manufacturer, is not guaranteed or endorsed by the publisher.
